# Study protocol for developing, piloting and disseminating the PRISMA-COSMIN guideline: a new reporting guideline for systematic reviews of outcome measurement instruments

**DOI:** 10.1186/s13643-022-01994-5

**Published:** 2022-06-13

**Authors:** Ellen B. M. Elsman, Nancy J. Butcher, Lidwine B. Mokkink, Caroline B. Terwee, Andrea Tricco, Joel J. Gagnier, Olalekan Lee Aiyegbusi, Carolina Barnett, Maureen Smith, David Moher, Martin Offringa

**Affiliations:** 1 Epidemiology and Data Science, Amsterdam UMC location Vrije Universiteit Amsterdam, de Boelelaan 1117, Amsterdam, Netherlands; 2 Quality of Care, Amsterdam Public Health, Amsterdam, The Netherlands; 3 Child Health Evaluative Sciences, The Hospital for Sick Children Research Institute, Toronto, Ontario Canada; 4 Department of Psychiatry, Faculty of Medicine, University of Toronto, Toronto, Ontario Canada; 5 Methodology, Amsterdam Public Health, Amsterdam, The Netherlands; 6 Li Ka Shing Knowledge Institute, St. Michael’s Hospital, Unity Health Toronto, Toronto, Ontario Canada; 7 Epidemiology Division and Institute of Health Policy, Management, and Evaluation, Dalla Lana School of Public Health, University of Toronto, Toronto, Ontario Canada; 8 Queen’s Collaboration for Health Care Quality Joanna Briggs Institute Centre of Excellence, Queen’s University, Kingston, Canada; 9 Department of Epidemiology and Biostatistics, Schulich School of Medicine and Dentistry, London, Ontario Canada; 10 Department of Surgery, Schulich School of Medicine and Dentistry, London, Ontario Canada; 11 Department of Orthopaedic Surgery, University of Michigan, Ann Arbor, Michigan USA; 12 Department of Epidemiology, School of Public Health, University of Michigan, Ann Arbor, Michigan USA; 13 Centre for Patient Reported Outcomes Research, Institute of Applied Health Research, University of Birmingham, Birmingham, UK; 14 Division of Neurology, Department of Medicine, University Health Network and University of Toronto, Toronto, Ontario Canada; 15 Cochrane Consumer Network, London, UK; 16 Centre for Journalology, Clinical Epidemiology Program, Ottawa Hospital Research Institute, Ottawa, Ontario Canada; 17 Management and Evaluation, Institute of Health Policy, University of Toronto, Toronto, Ontario Canada; 18 Division of Neonatology, The Hospital for Sick Children, Toronto, Ontario Canada

**Keywords:** COSMIN, PRISMA, Reporting guideline, Delphi study, Systematic review, Consensus, Outcome measurement instruments

## Abstract

**Background:**

Systematic reviews of outcome measurement instruments are important tools in the evidence-based selection of these instruments. COSMIN (COnsensus-based Standards for the selection of health Measurement INstruments) has developed a comprehensive and widespread guideline to conduct systematic reviews of outcome measurement instruments, but key information is often missing in published reviews. This hinders the appraisal of the quality of outcome measurement instruments, impacts the decisions of knowledge users regarding their appropriateness, and compromises reproducibility and interpretability of the reviews’ findings. To facilitate sufficient, transparent, and consistent reporting of systematic reviews of outcome measurement instruments, an extension of the PRISMA (Preferred Reporting of Items for Systematic reviews and Meta-Analyses) 2020 guideline will be developed: the PRISMA-COSMIN guideline.

**Methods:**

The PRISMA-COSMIN guideline will be developed in accordance with recommendations for reporting guideline development from the EQUATOR (Enhancing the QUAlity and Transparency Of health Research) Network. First, a candidate reporting item list will be created through an environmental literature scan and expert consultations. Second, an international Delphi study will be conducted with systematic review authors, biostatisticians, epidemiologists, psychometricians/clinimetricians, reporting guideline developers, journal editors as well as patients, caregivers, and members of the public. Delphi panelists will rate candidate items for inclusion on a 5-point scale, suggest additional candidate items, and give feedback on item wording and comprehensibility. Third, the draft PRISMA-COSMIN guideline and user manual will be iteratively piloted by applying it to systematic reviews in several disease areas to assess its relevance, comprehensiveness, and comprehensibility, along with usability and user satisfaction. Fourth, a consensus meeting will be held to finalize the PRISMA-COSMIN guideline through roundtable discussions and voting. Last, a user manual will be developed and the final PRISMA-COSMIN guideline will be disseminated through publications, conferences, newsletters, and relevant websites. Additionally, relevant journals and organizations will be invited to endorse and implement PRISMA-COSMIN. Throughout the project, evaluations will take place to identify barriers and facilitators of involving patient/public partners and employing a virtual process.

**Discussion:**

The PRISMA-COSMIN guideline will ensure that the reports of systematic reviews of outcome measurement instruments are complete and informative, enhancing their reproducibility, ease of use, and uptake.

**Supplementary Information:**

The online version contains supplementary material available at 10.1186/s13643-022-01994-5.

## Background

Outcome measurement instruments (OMIs) are important tools in clinical practice and research to monitor patients’ health status and in the evaluation of treatment efficacy, effectiveness, and safety [1, 2]. OMIs include patient questionnaires, assessments by health professionals, biomarkers, clinical rating scales, imaging or laboratory tests, and performance-based tests. Choosing the appropriate OMI for a specific construct, target population, and setting can be difficult and time consuming as there are often numerous OMIs, of uncertain qualities, that aim to measure the same construct and were developed for the same patient population [3–6]. Apart from the OMI’s quality (i.e., its measurement properties), feasibility of the OMI and interpretability aspects need to be considered. The measurement properties concern nine different aspects of reliability, validity, and responsiveness (Table 1), which are all important for OMIs with an evaluative application [7].

**Table 1:** COSMIN definitions of domains, measurement properties and aspects of measurement properties [7]

Domain	Term		Definition
	Measurement property	Measurement property aspect	
Reliability			The degree to which the measurement is free from measurement error
Reliability (extended definition)			The extent to which scores for patients who have not changed are the same for repeated measurement under several conditions: e.g., using different sets of items from the same OMI (internal consistency); over time (test-retest); by different persons on the same occasion (inter-rater); or by the same persons on different occasions (intra-rater)
	Internal consistency		The degree of interrelatedness among the items
	Reliability		The proportion of the total variance in the measurements which is due to ‘true’ differences between patients
	Measurement error		The systematic and random error of a patient’s score that is not attributed to true changes in the construct to be measured
Validity			The degree to which an OMI measures the construct(s) it purports to measure
	Content validity		The degree to which the content of an OMI is an adequate reflection of the construct to be measured
		Face validity	The degree to which (the items of) an OMI indeed seems to be an adequate reflection of the construct to be measured
	Construct validity		The degree to which the scores of an OMI are consistent with hypotheses (e.g., with regard to internal relationships, relationships to scores of other OMIs, or differences between relevant groups) based on the assumption that the OMI validly measures the construct to be measured
		Structural validity	The degree to which the scores of an OMI are an adequate reflection of the dimensionality of the construct to be measured
		Hypotheses testing	Idem construct validity
		Cross-cultural validity	The degree to which the performance of the items on a translated or culturally adapted OMI are an adequate reflection of the performance of the items of the original version of the OMI
	Criterion validity		The degree to which the scores of an OMI are an adequate reflection of a gold standard
Responsiveness			The ability of an OMI to detect change over time in the construct to be measured
	Responsiveness		Idem responsiveness
Interpretability^a^			The degree to which one can assign qualitative meaning (i.e., clinical or commonly understood connotations) to an OMI’s quantitative scores or change in scores

*COSMIN* COnsensus-based Standards for the selection of health Measurement INstruments

^a^Not considered a measurement property, but an important characteristic of a measurement instrument

Because of the widespread availability of many different OMIs for a given construct and population, systematic reviews in which the measurement properties of these OMIs are being evaluated and compared are increasingly being published (~ 140 new additions per year in the COSMIN database of Systematic Reviews since 2017) in order to facilitate their appraisal and selection [8]. By evaluating and summarizing the measurement properties reported for individual instruments, these systematic reviews are important tools in the evidence-based selection of OMIs. The COSMIN (COnsensus-based Standards for the selection of health Measurement INstruments) initiative has developed comprehensive and widely used guidance documents on how to conduct systematic reviews of OMIs [9–11]. Conducting a systematic review of OMIs for a specific context (e.g., construct, population) using the COSMIN methodology involves (1) systematically searching for the primary empirical studies that evaluate measurement properties and/or aspects of feasibility and interpretability; (2) appraising the methodological quality (i.e., risk of bias) of the included studies [12, 11]; (3) applying criteria for good measurement properties [13, 14]; and (4) summarizing the body of evidence and grading the quality of the evidence. With this combined information, recommendations can be formulated on whether or not to use an OMI.

Although the COSMIN guideline provides detailed guidance on how to conduct a systematic review of OMIs, key information is often missing in published systematic reviews [15–17]. For example, a previous study evaluating the quality and reporting of 246 systematic reviews of OMIs found that the syntax for the search strategy was lacking in more than half of the reports, and there was large variability in reporting of the appraisal process used for measurement properties [16]. In another study evaluating the quality of 102 reviews, it was unclear for 62% of them whether two reviewers evaluated the quality of the instruments. Moreover, in most reports the results from multiple studies on the same OMI were not synthesized at all (58%), or the methods to do so were not clearly described (22%) [17]. Lacking key information in published reports hinders the appraisal of the quality of OMIs, and might impact the decisions of knowledge users (e.g., researchers, healthcare providers, patients and policy-makers, who all rely on the findings of such systematic reviews) regarding the appropriateness of an OMI for a specific context [18].

Reporting guidelines and standards have been developed to help improve the completeness and transparency of different types of studies, data, and outcomes (e.g. [19–24],). With respect to OMIs, several guidelines exist, mostly focusing on patient-reported outcomes, such as guidelines for reporting results of quality of life assessments or patient-reported outcomes in clinical trials [25–27]. Large organizations have also published guidelines related to the use of patient-reported outcome measures (PROMs) in research, such as the Food and Drug Administration (FDA) [28], International Society of Quality of Life (ISOQOL) [29, 30], Outcome Measures in Rheumatology (OMERACT) [31], and others [32, 33]. Their guidelines often detail how to select PROMs or how to evaluate PROMs, but do not give extensive guidance on reporting of systematic reviews of PROMs, nor do they apply to other types of OMIs. With respect to systematic reviews, the PRISMA (Preferred Reporting of Items for Systematic reviews and Meta-Analyses) guideline [23] and its extensions (e.g. [34–37],) are best known for providing reporting guidance. The Institute of Medicine also published standards for reporting systematic reviews [38], which are a synthesis of standards from various organizations [23, 39–42]. Moreover, the Joanna Briggs Institute has published a manual for systematic reviews of measurement properties [43, 44], which endorses the PRISMA guideline [23] and COSMIN [12]. However, none of these guidelines specifically describes best-practices for the reporting of systematic reviews in which measurement properties of OMIs are assessed.

In 2021, the COSMIN reporting guideline for studies on measurement properties of PROMs has been developed [45]. This reporting guideline can improve and direct the reporting of *primary studies* investigating measurement properties of a PROM, but is not intended to direct the reporting of *systematic reviews* of PROMs or other types of OMIs. Thus far, authors of systematic reviews of OMIs have been encouraged to complete and adhere to the widely used generic version of the PRISMA guideline while reporting the results of their systematic review [23]. Yet, even though PRISMA captures some key aspects that are also included in the review process of OMIs (e.g., describing the search strategy), it does not include essential information specific and necessary to systematic reviews of OMIs, such as detailed information on the construct, population, type of OMI and measurement properties of interest, and methods used to appraise the methodological quality of the included studies and to evaluate the measurement properties of instruments. This information is required to make systematic reviews of OMIs reproducible and interpretable. Additionally, some (components of) items of the PRISMA 2020 checklist seem to be of limited relevance to systematic reviews of OMIs. Therefore, often peer reviewers and journal editors cannot properly appraise how a submitted review was done because key information is lacking, which contributes to the ongoing introduction of poor-quality reviews into the literature. Thus, there is a need for guidance regarding the reporting of systematic reviews of OMIs [18]. Extension and tailoring of the PRISMA 2020 guideline with key methodological details relevant to systematic reviews of OMIs ensures that reports of these reviews are comprehensive and informative, and facilitate their ease of use and uptake.

This protocol outlines the development process for an internationally harmonized reporting guideline for systematic reviews of OMIs, called the PRISMA-COSMIN guideline. Through an evidence-based and consensus-based process, PRISMA-COSMIN will evaluate what constitutes sufficient reporting of systematic reviews of OMIs employing COSMIN systematic review methodology.

## Methods

**Fig. 1:**
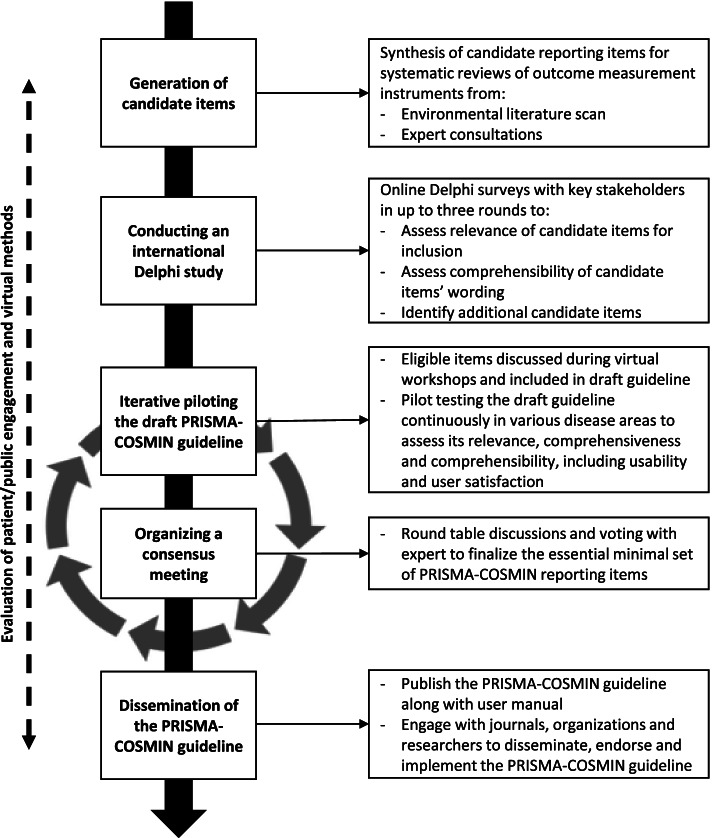
Outline of the PRISMA-COSMIN development process

The PRISMA-COSMIN guideline will be developed in accordance with recommendations for reporting guideline development from the EQUATOR (Enhancing the QUAlity and Transparency Of health Research) Network [46]. The development process for the PRISMA-COSMIN guideline consists of the following phases, depicted in Fig. 1: (1) generation of candidate reporting items through an environmental scan of the literature and expert consultations; (2) conducting an international Delphi study to assess candidate items’ relevance for inclusion, comprehensibility and wording, and to identify any key missing reporting items; (3) iterative piloting of the draft PRISMA-COSMIN guideline to assess its relevance, comprehensiveness and comprehensibility, along with usability and user satisfaction; and (4) organizing a consensus meeting to finalize the essential minimal set of PRISMA-COSMIN reporting items. An innovative methodology for reporting guideline development will be explored, by employing a completely virtual process and including patients, caregivers, and members of the public as research partners (hereafter referred to as patient/public partners) throughout the project, which is not yet a formal part of the EQUATOR guideline. Patient/public engagement and acceptability of virtual methods will be evaluated during and after the project, resulting in an overview of barriers and facilitators of this innovative methodology for reporting guideline development.

### Project launch, steering committee, and technical advisory group

PRISMA-COSMIN was registered on the EQUATOR Network library on 22 September 2020, and officially launched in October 2021 after funding for development of the PRISMA-COSMIN guideline was secured from the Canadian Institutes of Health Research (CIHR).

The steering committee for the PRISMA-COMSIN guideline consists of eight experienced researchers with collective international expertise in reporting guideline development, knowledge synthesis and translation, and measurement science, including representation from COSMIN and PRISMA. A patient/public partner (MS) with expertise in patient/public engagement is also part of the steering committee. The steering committee will lead the study and provide project oversight. They will prepare and provide all necessary materials for each of the phases. They will generate candidate items, design the international Delphi study, and organize the consensus meeting. They will not act as panelists in the Delphi study but have the authority to make final decisions in each project phase, including a vote in the consensus meeting.

The steering committee will be supported by a 10-member technical advisory group, composed of key outcome methodologists from international leading groups in measurement science and reporting standards (see Additional file 1 for members of the steering committee and technical advisory group). The technical advisory group will be asked to give feedback on each stage of the project, including the opportunity to give feedback on the study protocol (this manuscript), to review the initial draft version of the checklist, to contribute to the Delphi study as panelists, to contribute to piloting the PRISMA-COSMIN checklist as pilot testers, to participate in the consensus meeting, and to give feedback on the (draft) guideline and user manual. Because of their large, international network, members of the steering committee and the technical advisory group will also be able to identify other panelists as well as other pilot testers, and other consensus meeting experts.

### Phase 1: Generation of candidate items

To generate candidate items, the PRISMA 2020 checklist [23] was used as a starting point and its reporting items were evaluated for applicability for systematic reviews evaluating OMIs by the steering committee. If deemed applicable, the reporting item was refined to include aspects specific to systematic reviews of OMIs when necessary. The COSMIN guideline for systematic reviews [9, 10] and the COSMIN reporting guideline for studies on measurement properties of PROMs [45] served as guidance documents in this process. New items were added as well. Next, an environmental scan of the literature was conducted to search for scientific articles and existing guidelines that describe reporting items of OMIs or systematic reviews. Literature search results from previous reporting guideline development projects were used [45, 47], and in addition, a search was conducted for reporting recommendations of systematic reviews of OMIs. Reporting recommendations extracted from identified guidance documents [9, 10, 13, 14, 19–33, 38–44, 12, 45, 34–37] were compared to the candidate reporting items to support, refute and refine them, and to identify additional reporting items, in order to arrive at a comprehensive item list.

The comprehensive item list, containing all possible reporting items, was applied by a member of the steering committee (EE) to three existing high-quality systematic review reports: (1) a systematic review of *all PROMs* measuring physical functioning in type 2 diabetes, co-authored by some members of the steering committee [48]; (2) a systematic review of *one specific PROM* (i.e., the Dutch-Flemish PROMIS physical function item bank) [49]; and (3) a systematic review of *digital monitoring devices* measuring oxygen saturation and respiratory rate in COPD [50]. By applying the comprehensive item list to three different types of systematic reviews, a distinction could be made between items applicable for all review types, and items applicable to certain review types. Based on the results, the comprehensive item list, containing all possible items, was synthesized in an optimal item list, containing essential items. At this time point in the project, the current protocol paper was submitted for publication.

A table detailing all results thus far, including all possible reporting items, the results from the three systematic reviews, and the essential reporting items, will be presented to the entire steering committee and technical advisory group. Following these expert consultations, the preliminary PRISMA-COSMIN checklist will be iteratively presented and modified (i.e., new items will be added and existing items will be modified) based on feedback from the experts obtained in videoconference meeting and/or email communications.

### Phase 2: International Delphi study

An online international Delphi study with a web-based questionnaire will be conducted. A Delphi study is a procedure that can be used to generate debate and to structure and organize group communication processes. It is an iterative, multistage process for making the best use of all available information, through structured rounds of surveys interspersed by controlled feedback, to arrive at consensus of opinion among a panel of stakeholders [51]. Based on the results of the generation of candidate items, described above, a list of reporting items, together with operational definitions and examples, will be developed for use in the Delphi. The complete questionnaire for the first Delphi round, including invitation texts, will be designed and pilot tested with five individuals from the steering committee and/or technical advisory group. Feedback on the design will be collected and the questionnaire will be revised accordingly.

#### Recruitment of panelists

International key stakeholders involved in the design, conduct, publication, and/or application of systematic reviews of OMIs will be invited to be panelists in the Delphi study. Panelists will be selected to represent various scientific backgrounds (e.g., clinical medicine, biostatistics, psychometrics/clinimetrics, epidemiology) as well as leading relevant organizations (e.g., ISOQOL, OMERACT, Cochrane, ISPOR) and journals (e.g., Quality of Life Research, Journal of Patient-Reported Outcomes, Journal of Clinical Epidemiology). In addition, patient/public partners (*n* = 5 in total) will be invited to contribute to the Delphi study.

Relevant groups, organizations, and individuals will be identified by the steering committee and technical advisory group through their professional contacts, networks, and affiliations. Known panelists from other relevant Delphi studies (e.g. [52, 11, 45],), as well as authors from relevant guidance documents (e.g. [12, 38, 29],) and authors who have conducted multiple systematic reviews of OMIs (as identified through the COSMIN database for systematic reviews [8]) will be invited. Patient/public partners will be recruited through newsletters, social media channels, and contact persons of relevant organizations that often involve them (e.g., OMERACT’s Patient Research Partner Network, COMET’s PoPPIE working group, Cochrane Consumers Network). Invitation to the Delphi study will include text asking candidate panelists to forward the invitation to other qualified colleagues or relevant groups or organizations that might be interested to contribute. There will be no geographical restrictions on eligibility. The Delphi study will be conducted in English. Panelists can enter the study in each round, but retention between rounds will be encouraged through communications conveying the importance of completing the entire Delphi study [53]. Panelists who complete the entire Delphi study will receive an acknowledgement in publications for their contributions, with their permission.

A minimum of 30 panelists will be considered appropriate [54]. Based on previous experiences [45, 11], it is anticipated that at the most 50% of the invited persons will complete at least one round. Therefore, initially at least 150 individuals will be invited. Those willing to be panelists will be asked to provide informed consent and complete a brief registration form, including questions regarding basic demographic information, such as job title, country of work, work setting, scientific background, and relevant work experience. Panelists from the patient/public community will be asked to identify their relevant patient/public engagement or lived experience. If less than 60 people are willing to be panelists, more persons will be invited, until 60 have agreed to complete round 1. Maximum variation with respect to panelists scientific background and type of work experience will be sought; recruitment strategies will be adjusted if certain groups are under- or overrepresented.

#### Delphi procedure

Virtual, interactive onboarding sessions with patient/public partners will be held approximately 3 weeks before the Delphi survey launch. These onboarding sessions will facilitate shared clarity and agreement on methodological and practical challenges in conducting systematic reviews of OMIs. In these onboarding sessions, the project will be explained and patient/public partners will be prepared for involvement in the Delphi study. It will also be determined on which topics they may need additional background reading and support. The onboarding sessions will ensure that patient/public partners have understandable information, so that they can meaningfully contribute to the Delphi study. The number and exact content of the onboarding sessions will be determined after meeting with patient/public partners, once their knowledge level and needs have been identified.

Prior to the Delphi study, all panelists will be given information about the objectives and process of the Delphi study. A web-based survey will be designed using Research Electronic Data Capture (REDCap) software [55]. Each Delphi round will be open for approximately 3 weeks. Depending on the results, we expect that we will need up to three rounds, to ensure that all items are evaluated twice. Panelists will receive weekly reminders approximately 1 week after the launch of each Delphi round. The responses of the panelists will remain anonymous throughout the Delphi study and will be analyzed anonymously as well; only delegate members of the steering committee will have access to the identifiable responses. Completion of each Delphi round will be voluntary.

**Fig. 2:**
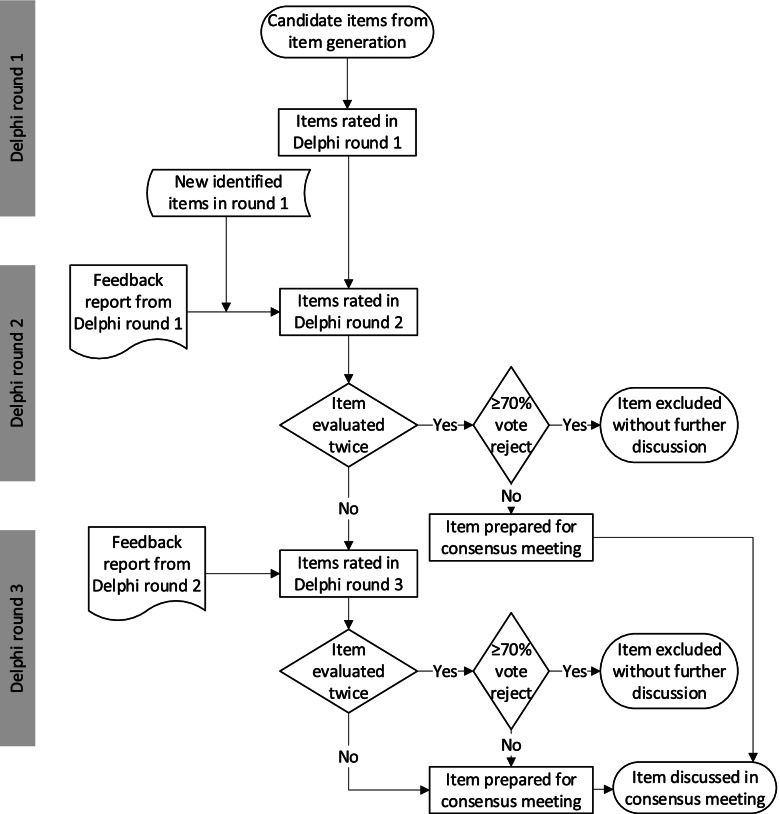
Outline of the Delphi study process

Figure 2 outlines the Delphi study process. Panelists will be asked to rate each reporting item identified in the generation of candidate items for inclusion in the PRISMA-COSMIN guideline. The presentation of items will be such that it will be clear whether an item is new (i.e., not present in the PRISMA 2020 checklist), modified (i.e., covered in part in the PRISMA 2020 checklist), or existing (i.e., already present in the PRISMA 2020 checklist). Panelists will be asked to rate the relevance for inclusion of each item on a 5-point scale (1—defintely reject, 2—probably reject, 3—neutral, 4—probably keep, 5—definitely keep). Panelists can also opt for “not my expertise” to accommodate those who do not have the level of expertise required to rate the item. This option may be especially relevant to reassure patient/public partners that it is not necessary for them to rate all items on the list. Panelists will be encouraged to provide a rationale for their ratings, to suggest modifications of definitions or wording of items, to indicate any possible overlap between items or possibilities to aggregate items, and to suggest potential new items not included in the list. Suggestions for item modification, aggregation or new items will be deliberated and discussed by the steering committee, and integrated into round 2. All findings will be organized into a feedback report that will be sent to the panelists.

In Delphi round 2, panelists will receive the feedback report containing the results of round 1 presented both quantitatively (i.e., the distribution of ratings/degree of consensus as well as their own rating) and qualitatively (i.e., the suggestions and comments of the panelists regarding each item). Panelists will again be asked to rate the relevance of each of the reporting items, as well as newly identified items, for inclusion in the PRISMA-COSMIN guideline on the same scale. They will again be encouraged to provide a rationale for their ratings, to suggest modifications of definitions or wording of items, and to indicate any possible overlap between items or possibilities to aggregate items. They will no longer be actively asked to suggest potential new items not included in the list. If an item is evaluated for the second time, consensus for exclusion of an item will be reached if at least 70% of the panelists opt for reject (i.e., score 1 or 2). These items will be considered confirmed for exclusion from the PRISMA-COSMIN guideline and will not be further discussed. All other items originating from the generation of candidate items (i.e., items that are evaluated twice) will move forward to the consensus meeting. Any new items proposed in round 1 will move forward to round 3. All findings will again be organized into a feedback report, containing similar information as the first feedback report.

In Delphi round 3, panelists will receive the feedback report containing the results of round 2. The list of items already considered confirmed for exclusion from the PRISMA-COSMIN guideline or prepared for the consensus meeting will also be provided. Panelists will again be asked to rate the relevance of each of the remaining reporting items for inclusion in the PRISMA-COSMIN guideline, on the same scale. Panelists will also be encouraged to provide a rationale for their ratings, to suggest modifications of definitions or wording of items, and to indicate any possible overlap between items or possibilities to aggregate items. They will not be actively asked to suggest potential new items not included in the list. As in round 2, consensus for exclusion of an item will be reached if at least 70% of the panelists opt for reject (i.e., score 1 or 2), and these items will be considered confirmed for exclusion from the PRISMA-COSMIN guideline and will not be discussed further. All other items will move forward to the consensus meeting.

### Phase 3: Piloting of the PRISMA-COSMIN guideline

Items that will move forward to the consensus meeting will be discussed during a series of virtual workshops with members of the steering committee and technical advisory group. Phrasing of items will be clarified as necessary, considering the feedback of panelists from the Delphi study. Relevant content for the user manual, containing the background, rationale and justification for each reporting item, together with examples of good reporting, will also be discussed. The steering committee will develop a draft PRISMA-COSMIN guideline along with draft user manual.

Piloting the PRISMA-COSMIN guideline and user manual will be an iterative process, starting after the Delphi study and continuing till after the consensus meeting, to pilot test each subsequent version of the PRISMA-COSMIN guideline and user manual. Researchers and clinicians in various disease areas (e.g., mental health, rheumatology, surgery, child health) with expertise in systematic reviews of OMIs will be asked to pilot test the draft PRISMA-COSMIN guideline along with the user manual, to ensure the PRISMA-COSMIN guideline is applicable to all fields and outcome types. For each disease area, the PRISMA-COSMIN guideline will be applied to at least four different systematic reviews of OMIs. Pilot testers (*n* = 4 per disease area) will be selected by the steering committee and technical advisory group, and an open invitation for pilot testing and feedback will be distributed through social media channels and newsletters of relevant organizations.

The relevance and comprehensibility of each reporting item, and the comprehensiveness of the PRISMA-COSMIN guideline will be evaluated on a 7-point scale (1 = not at all to 7 = to a great extent). Pilot testers can also leave comments and suggestions. In addition, usability and user satisfaction with the PRISMA-COSMIN guideline will be determined by three questions:

1. Is the PRISMA-COSMIN guideline user friendly? Why (not)?
2. How much time does it take to complete the guideline?
3. Will it help or hinder the report writing process? Why?

The qualitative and quantitative feedback from these pilot tests will be incorporated to improve the quality of the PRISMA-COSMIN guideline and the user manual by the steering committee.

### Phase 4: Consensus meeting

A consensus meeting will be organized in Toronto, Canada, to obtain expert consensus on which items will be included with their finalized wording in the final PRISMA-COSMIN guideline. Besides the steering committee and technical advisory group, editors of selected journals, members of important organizations, patient/public partners, and Delphi panelists with appropriate expertise will be invited, in order to obtain 20–25 diverse experts. Experts not able to attend in-person can join through videoconference.

Each candidate PRISMA-COSMIN item will be presented, along with results from the Delphi study, the recommendations from the virtual workshops and the results from the pilot tests. Moderated round table discussions for each item will follow, after which anonymous voting on each item will be conducted. Voting options are “include in final guideline”, “exclude from final guideline”, “merge with other item”, or “unsure”. Consensus for inclusion or exclusion of an item in/from the final guideline will be reached if at least 70% of the experts vote for inclusion or exclusion, respectively. Items that do not reach consensus will be subject to another round table discussion and voting procedure. This process will continue until all items have reached consensus. Round tables discussions will be audio recorded. If consensus for some items will not be reached before the end of the meeting, the final decision will be made by the steering committee, taking into account the statements from the round table discussions.

### Publication and dissemination

Throughout the project, active presence on social media (e.g., Twitter, LinkedIn) and relevant websites (e.g., the COSMIN website [56]) will be maintained. The methods and preliminary results of the project will be presented at international, national, and local conferences. To disseminate the results of the project, a final PRISMA-COSMIN guideline with user manual will be developed by the steering committee and the project will be presented at relevant conferences. In addition, a scientific manuscript detailing the process of the PRISMA-COSMIN guideline development will be published in an open access journal. The PRISMA-COSMIN guideline and user manual will be made freely available through relevant websites, such as the EQUATOR website [57], the PRISMA website [58], and the COSMIN website [56]. Important journals and organizations currently endorsing the PRISMA guideline will be approached to also endorse the PRISMA-COSMIN guideline. Other dissemination strategies established throughout the project will also be considered.

### Evaluation of innovative methods

To evaluate whether the methods for reporting guideline development (i.e., involving patient/public partners and employing a virtual process) have contributed to enhanced engagement and increased fidelity, contributors’ satisfaction will be monitored at all project phases (Table 2).

**Table 2:** Overview of proposed evaluation instruments administered to stakeholders at different phases of the project

	Public and Patient Engagement Evaluation Tool [59, 60]				Patient Engagement In Research Scale [61]	Modified Acceptability E-scale [62]	PANELVIEW instrument [63]
	Patient-partner questionnaire	Project coordinator questionnaire					
	One-time engagements	Planning engagement	Assessing engagement	Assessing impact of engagement			
Prior to onboarding sessions		Steering committee					
After onboarding sessions	Patient/public partners		Steering committee			Patient/public partners	
After Delphi study					Patient/public partners	Panelists	Panelists
After virtual workshops						Steering committee and technical advisory group	
After consensus meeting			Steering committee		Patient/public partners		Consensus meeting experts
Three months after consensus meeting				Steering committee			

The Patient Engagement In Research Scale (PEIRS) [61] and the Public and Patient Engagement Evaluation Tool (PPEET) [59, 60] will be used to evaluate patient/public engagement. The PEIRS is a 37-item questionnaire that can be used to quantify meaningful patient engagement in research [61]. Patient/public partners will be asked to complete PEIRS after the Delphi study and the consensus meeting. The PPEET consists of a series of questionnaires for patient/public partners, project coordinators and managers of organizations [59, 60], of which the first two (i.e., the patient partner and project coordinator questionnaire) will be used. Patient/public partners will be asked to complete the patient partner questionnaire ‘one-time engagements’ module after the onboarding sessions. Each member of the steering committee will be asked to complete the project questionnaire ‘planning the engagement’ module prior to the onboarding sessions, the ‘assessing the engagement’ module after the onboarding sessions and the consensus meeting, and the ‘assessing the impact of engagement’ module once the project is finalized, three months after the consensus meeting. In addition to these instruments, a focus group discussion (or individual interview, if preferred) will be held with patient/public partners and members of the steering committee after the project to qualitatively assess patient engagement (e.g., what went well, what could be improved, what are lessons learned for future methodological projects).

The virtual methods employed (i.e., the onboarding sessions, the Delphi survey, and the virtual workshops) will be evaluated among all activity participants after the respective activity with a modified version of the Acceptability E-scale [62]. The Acceptability E-scale is a 6-item scale measuring the acceptability and usability of computer-based assessments or programs. A summary score of 24 or higher (i.e., ≥ 80% of the highest possible score) will be used as a threshold, indicating that the computer-based assessment is acceptable to users [62].

The overall guideline development process will be evaluated with the PANELVIEW instrument, which assesses contributors’ satisfaction with and perceived appropriateness of the development of the PRISMA-COSMIN guideline [63]. Panelists of the Delphi study will be asked to complete the PANELVIEW instrument after the Delphi study, whereas experts in the consensus meeting will be asked to complete the PANELVIEW instrument after the consensus meeting.

A scientific research methods manuscript will be published regarding the evaluation of the patient/public engagement and the virtual process, barriers and facilitators encountered, and lessons learned, to direct future reporting guideline developers.

## Discussion

The PRISMA-COSMIN guideline will provide guidance on what should be minimally reported in systematic reviews of OMIs. The development and implementation of the PRISMA-COSMIN guideline aims to harmonize and standardize the reporting of systematic reviews of OMIs and ensure reports are comprehensive. This will make studies reproducible, contributes to the transparency of the conclusions drawn, and reduces research waste. Most importantly, it allows end-users of systematic reviews to formulate their own conclusions.

The evidence-based and consensus-based processes that will be used in the development of the PRISMA-COSMIN guideline will contribute to the acceptance and uptake of the PRISMA-COSMIN guideline by journals, organizations and individual researchers. Guidelines developed by individual experts or small research groups often do not have sufficient credibility to be accepted and implemented. Through involvement of a large group of experts with different scientific backgrounds, representing key international organizations, the PRISMA-COSMIN guideline has a good chance to become widely used.

Including patient/public partners and employing a virtual process are relatively novel methods in the field of reporting guideline development. Patients and members of the public can be considered direct end-users of the results of systematic reviews of OMIs, as the conclusions (i.e., the recommendation to use a specific OMI) can impact them directly. Therefore, and since PROMs are used increasingly, including the perspective of patient/public partners on what is relevant to report and how it should be reported is important. We recognize the challenge of engaging with patient/public partners on this complex methodological topic and have built in supports to enable them to participate meaningfully. We also note that the use of virtual methods for reporting guideline development might not be as challenging as it would have been pre COVID times. Due to the COVID pandemic, new patterns of international collaboration, exchange of ideas and electronic participation have emerged [64], which we anticipate to be beneficial for the development of the PRISMA-COSMIN guideline. Extensive evaluation of both these relatively novel methods will result in the identification of barriers and facilitators, and lessons learned. Evaluation results will be shared through a scientific research methods manuscript, to direct future reporting guideline developers who want to employ similar methods.

## Conclusions

Systematic reviews of OMIs’ measurement properties are important tools in the evidence-based selection of these instruments, and are increasingly being published [8]. However, key information is often missing from published reports [15–17], compromising reproducibility and interpretability. Development of the PRISMA-COSMIN guideline will ensure that the reports of systematic reviews of OMIs are complete and informative, and include patient/public partners’ perspectives, thus enhancing their ease of use and uptake. We expect the final version of the PRISMA-COSMIN guideline and user manual to be ready in the last quarter of 2023.

## Supplementary Information

**Additional file 1.** Group membership for the PRISMA-COSMIN guideline

## Data Availability

Not applicable

## Abbreviations

CIHR: Canadian Institutes of Health Research
COSMIN: COnsensus-based Standards for the selection of health Measurement INstruments
EQUATOR: Enhancing the QUAlity and Transparency Of health Research
FDA: Food and Drug Administration
ISOQOL: International Society of Quality of Life
OMERACT: Outcome Measures in Rheumatology
OMI: Outcome measurement instrument
PEIRS: Patient Engagement In Research Scale
PPEET: Public and Patient Engagement Evaluation Tool
PRISMA: Preferred Reporting of Items for Systematic reviews and Meta-Analyses
PROM: Patient-reported Outcome Measure
