# Supplementary material for: Study protocol for developing, piloting and disseminating the PRISMA-COSMIN guideline: a new reporting guideline for systematic reviews of outcome measurement instruments
Source: Syst Rev. 2022 Jun 13;11:121. doi: 10.1186/s13643-022-01994-5 (PMC9195229; doi:10.1186/s13643-022-01994-5)
Supplement: Supplementary file 1 — Additional file 1. Group membership for the PRISMA-COSMIN guideline [file 13643_2022_1994_MOESM1_ESM.pdf]

Additional file 1. Group membership for the PRISMA-COSMIN guideline

Name	PRISMA-COSMIN role	Primary affiliation
Ellen Elsman	Steering committee	Department of Epidemiology and Data Science, Amsterdam UMC, Vrije Universiteit Amsterdam, Amsterdam Public Health Research Institute, Amsterdam, the Netherlands
Martin Offringa	Steering committee	Child Health Evaluative Sciences, The Hospital for Sick Children Research Institute, Toronto, Ontario, Canada
Nancy Butcher	Steering committee	Child Health Evaluative Sciences, The Hospital for Sick Children Research Institute, Toronto, Ontario, Canada
David Moher	Steering committee	Centre for Journalology, Clinical Epidemiology Program, Ottawa Hospital Research Institute, Ottawa, Canada
Andrea Tricco	Steering committee	Li Ka Shing Knowledge Institute, St. Michael's Hospital, Unity Health Toronto, Toronto, Ontario, Canada
Wieneke Mokkink	Steering committee	Department of Epidemiology and Data Science, Amsterdam UMC, Vrije Universiteit Amsterdam, Amsterdam Public Health Research Institute, Amsterdam, the Netherlands
Caroline Terwee	Steering committee	Department of Epidemiology and Data Science, Amsterdam UMC, Vrije Universiteit Amsterdam, Amsterdam Public Health Research Institute, Amsterdam, the Netherlands
Joel Gagnier	Steering committee	Department of Epidemiology and Biostatistics, Schulich School of Medicine and Dentistry, London, Ontario, Canada
Maureen Smith	Steering committee	Cochrane Consumer Network, London, UK
Dorcas Beaton	Technical advisory group	Institute for Work & Health and Institute for Health Policy Management and Evaluation, University of Toronto, Toronto, Ontario, Canada
Olalekan Lee Aiyegbusi	Technical advisory group	Centre for Patient Reported Outcomes Research, Institute of Applied Health Research, University of Birmingham, Birmingham, United Kingdom
Cornelia Borkhoff	Technical advisory group	Institute of Health Policy, Management, and Evaluation, University of Toronto, Toronto, Ontario, Canada
Suneeta Monga	Technical advisory group	The Hospital for Sick Children, Toronto, Canada
Karen Wong	Technical advisory group	The Hospital for Sick Children, Toronto, Canada
Anne Klassen	Technical advisory group	McMaster University, Hamilton, ON, Canada
Carolina Barnett-Tapia	Technical advisory group	Division of Neurology, Department of Medicine, University Health network and University of Toronto, Toronto, Ontario, Canada
Karolin Krause	Technical advisory group	Evidence Based Practice Unit, Faculty of Brain Sciences, University College London, London, UK
Elizabeth Potter	Technical advisory group	School of Epidemiology and Public Health, University of Ottawa, Ontario, Canada
Peter Tugwell	Technical advisory group	Department of Medicine and School of Epidemiology, Public Health and Preventive Medicine, University of Ottawa, Ottawa, Ontario, Canada
